# The combined effect of Trigonella foenum seeds and Coriandrum sativum leaf extracts in alloxan-induced diabetes mellitus wistar albino rats

**DOI:** 10.6026/97320630015716

**Published:** 2019-10-22

**Authors:** Sree Sudha Tanguturi Yella, Raghupathi Niranjan Kumar, Chakali Ayyanna, Anjaly Mary Varghese, P Amaravathi, Yakaiah Vangoori

**Affiliations:** 1 Department of Pharmacology, AIIMS Medical Institute, Raipur, Chhattisgarh, India, 492099; 2 Department of Pharmacology, Santhiram College of Pharmacy, Nandyal, Andhra Pradesh, India, 518501; 3 Department of Pharmacology, CES College of pharmacy, Kurnool, Andhra Pradesh,India, 518001; 4 Department of Pharmacology, Santhiram Medical College and General Hospital, Nandyal, Andhra Pradesh, India, 518502

**Keywords:** Diabetes mellitus, alloxan, glibenclamide, Trigonella foenum, Coriandrum sativum

## Abstract

Diabetes mellitus is a group of heterogeneous disorders commonly presenting with episodes of hyperglycemia and glucose intolerance, as a result of lack of insulin, ineffective insulin action, and/or both. It is our interest to study the effect of ethanolic extract of Trigonella foenum seeds (fenugreek) and Coriandrum sativum leaves (dhaniya) or its combination in alloxan induced diabetes mellitus wistar albino rats. Rats were randomly separated into six groups where group 1 animals received 2% acacia, group 2 animals received alloxan dose of 150 mg/kg, group 3 animals received glibenclamide dose of 0.5 mg/kg and group 4, 5 and 6 animals received ethanolic extracts of Trigonella foenum seeds, Coriandrum sativum leaves and combination of both extracts at the dose of 100mg/kg for 21 days. Different biochemical parameters such as hepatic and renal biomarkers and histopathology of pancreas were studied. Combination of both extracts showed significant decrease in blood glucose, cholesterol, triglycerides, LDL, VLDL levels, SGOT, SGPT, urea, creatinine and increase in HDL levels and body weight than individual extracts. Thus, we show the antidiabetic activity of poly herbal formulation using biochemical and histo pathological data.

## Background

Herbals are helpful to mankind. A number of them are used for healing purpose. The importance of medicinal plants in drug discovery is highlighted by the World Health Organization (WHO). Such plants are in demand by pharmaceutical companies for their active ingredients [1,2]. Diabetes mellitus is a disorder affecting almost 6% of the world population and the dynamics of the diabetes are changing quickly in low-to middle-income countries [3]. It is known that 80% of the world diabetic population will be from low-and middle-income countries in 2030 as per the International Diabetes Federation's (IDF) estimates. It is one of the six major causes of death caused by various systemic problems. Diabetes mellitus is treated by hormone therapy (insulin) or by administering glucose-lowering agents such as alpha-glucosidase inhibitors, sulfonyl ureas, biguanides and thiazolidinediones. Expansion of an adverse event is one of the complications in the treatment of any systemic disorder. It is known that 10-25% of patients in the USA experience an adverse drug reaction and these adverse drug reactions are responsible for 3.4-7.0% of hospital admissions [5]. Hence, there is an interest for drug development with good therapeutic potential with less adverse events [4].

Many floras have been recognized for the treatment of various systemic disorders in traditional systems of medicine. Many of the traditional/indigenous systems of medicine are more effective than the modern system of medicine. However, they suffer from lack of complete standardization which is one of the important challenges faced by the traditional system of medicine. The concept of poly herbal formulation is well documented in the ancient literature. It has been realized that the poly herbal formulation has better and extended therapeutic potential than mono herbal treatments. Hence, it is of interest to formulate and evaluate the therapeutic effects of a poly herbal formulation using a combination of plant extracts having known antidiabetic activity in rodent in vivo models.

## Methodology

### Collection of seeds:

Taxonomically identified seeds of Trigonella foenum and Coriandrum sativum were collected from the Nandyal region, Kurnool district. The collected seeds were authenticated at the Department of Botany, SV University, Tirupati, Andhra Pradesh, India.

### Ethanolic extraction procedure:

The dried seed powders of Trigonella foenum and Coriandrum sativum were defatted by using n-hexane with maceration technique. The defatted powder is dried at room temperature. The dried defatted powder was then extracted with ethanol at 700c by soxhlet apparatus. The solvent in the extract was removed by distillation and dried to a solid mass.

### Preparation of poly herbal formulation:

The poly herbal formulation contained the ethanolic extracts of Trigonella foenum and Coriandrum sativum in the ratio of 1:1. The quality of the poly herbal formulation was tested as per the WHO guidelines for the quality control of herbal materials [6].

### Experimental animals:

Adult wister rats weighing about 150 to 180 g were used in the study. The study protocol was reviewed and approved by the institutional animal ethical committee of Santhiram Medical College, India. Animals were obtained from Sainath Enterprises, Hyderabad, India. Rats were housed in poly acrylic cages (38x23x10 cm). They were housed in an air-conditioned room and were kept in standard laboratory conditions under natural light and dark cycle (approximately 12 h light/ 12 h dark). The humidity maintained is 60±5% and an ambient temperature is 25±2%. All experiments were performed between 9:00 am to 4:00 pm. The animals were given free access to standard diet and water ad libitium and allowed to acclimatize for one week before the experiments.

### Drugs and chemicals used in the study:

We used n-hexane from MOLY Chem. India (P) Ltd, Mumbai, ethanol from Santhiram College of Pharmacy, alloxan NP from CHEM BOMBAY and several Kits (Glucose, LDL, TG and HDL) from Excel Diagnostics Pvt. Ltd. Hyderabad.

### Experimental induction of diabetes:

In this study, diabetes was induced by single intra peritoneal injection of alloxan (150mg/kg) [7]. The alloxan was prepared by dissolving 150 mg of alloxan in 1ml of normal saline solution. The animals were fasted over night and allowed to drink 5% glucose solution. Alloxan was given by intra peritoneal route in dose of 150 mg/kg to all rats except group-1 animals. Fasting plasma blood glucose was estimated after 72 hours of alloxan injection. Animals with plasma glucose of > 200 mg/dl were included in groups II-VI. The rats were divided into six groups consisting of six rats in each group followed by treatment for 21 days.

### Experimental design:

Animals were randomized and divided into five experimental groups (n=6) as follows.

Group 1: Normal was received vehicle (2% acacia, 10ml/Kg body weight, P.O.)

Group 2: Control was received alloxan (150mg/Kg, I.P)

Group 3: Standard was received alloxan (150mg/Kg, I.P) and Glibenclamide (0.5 mg/kg/P.O)

Group 4: Received alloxan (150mg/kg, i.p) and EETF (100 mg/Kg, P.O.)

Group 5: Received alloxan (150mg/kg, i.p) and EECS (100 mg/Kg, P.O.)

Group 6: Received alloxan (150mg/kg, i.p) and EETF (100 mg/kg, p.o.) + EECS (100 mg/kg/P.O.)

The doses were given for 21 days as multiple dose studies. Animals were provided with food and water as usual before experiment.

### Biochemical estimations:

The blood samples were drained on 7th, 14th and 21st day from the retro orbital venous plexus of rats under anesthesia using a glass capillary tube and the blood was centrifuged (2500 rpm/10min) to get serum. The serum thus obtained was used for biochemical estimations.

### Histo pathological analysis

Part of the pancreas tissue was potted in 10% formalin for 2 days. The pancreas was dehydrated with alcohol (later with 70, 80, 90%, and absolute alcohol) for 12 h each. The tissues were again cleaned by using xylene for 15-20 min and they were subjected to paraffin infiltration in automatic tissue processing unit. The tissue blocks were prepared and the blocks were cut using microtome to get sections of thickness 5µm. The sections were taken on a microscopic slide on which egg albumin was applied and allowed for drying. Finally, the sections were stained with eosin (acidic stain) and hemotoxylin (basic stain).

### Statistical analysis:

All the data were reported in mean ± SEM. The significance of variation in means between control and treated animals was determined by One-way analysis of variance (ANOVA) followed by Tukey's multiple comparison test (graph pad prism 5.03). P < 0.05 was considered statistically significant.

## Results

The diabetic animals showed considerable reduction in body weight when compared to the control animals throughout the study. However, the individual plants therapy, poly herbal formulation and glibenclamide reserved the diabetes induced body weight reduction. The results are presented in Table 1.

Diabetic control animals showed severe hyperglycemia compare to normal animals. The mean blood glucose level in the diabetic control group on day 1 was 206.25 ± 9.84 mg/dl and on day 21 was 318.66 ± 14.25mg/dl. It was observed that the standard drug glibenclamide lowered the blood glucose level significantly, bringing it back to near normal level, where as the ethanolic extract of Trigonella foenum seeds, Coriandrum sativum leaves (P < 0.001) and its poly herbal formulation (P < 0.001) at 100 mg/kg significantly decreased the fasting blood serum glucose level in the diabetic rats on 7th, 14th, and 21st days, as compared to the diabetic control group. The results are shown in Table 2.

Alloxan administered animals will raise the serum enzyme levels such as cholesterol, triglycerides, LDL, VLDL and decrease the HDL level, but Glibenclamide (0.5 mg/kg/P.O) and ethanolic extract of Trigonella foenum seeds, Coriandrum sativum leaves and its polyherbal formulation reversed the above alloxan induce changes. There was a significant decrease of cholesterol, triglycerides, LDL, VLDL and significant increase in HDL levels after 21 days in group 4, 5 and 6 animals when compare to group-2 animals (p < 0.001). But the groups 6 animals treated with polyherbal formulation showed better results than individual therapy and the results showed in Table 3, Table 4,Table 5,Table 6 and Table 7.

The diabetic rats showed significant (P < 0.001) increase in hepatic and renal biomarkers includes SGOT, SGPT, Urea and creatinine, whereas the levels in the treatment group remained within normal limits at the end of the study. Effects of herbal formulation and glibenclamide on the hepatic and renal biomarkers profile of diabetic animals were presented in Table 8.

**Figure 1:**
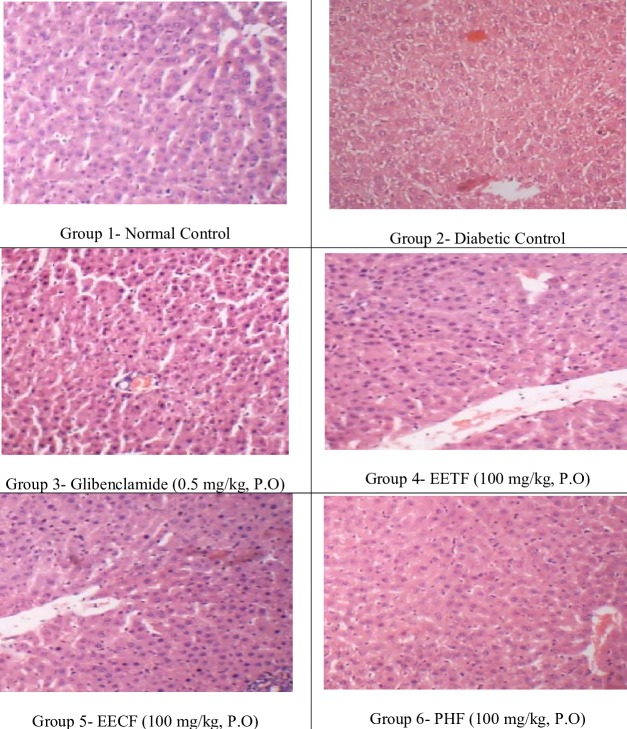
Histopathology of pancreas in rats

The histo pathological analysis of pancreas exposed severe congestion, giant decrease in the number of islets of Langerhans and β cells, fibrosis and inflammatory cell infiltration into the islets of Langerhans in alloxan induced hyper glycemic rats. Ethanolic extract of Trigonella foenum seeds, Coriandrum sativum leaves and its poly herbal formulation at the dose of 100mg/kg showed mild congestion and mild decrease in the number of islets of Langerhans with normal β cell population, indicating significant amount of recovery. Glibenclamide treatment showed moderate congestion with moderate decrease in the number of islets of Langerhans and β cells and mild lymphocytic infiltration were presented in Figure 1.

## Discussion

The poly herbal formulation was formulated using the ethanolic extracts of the seeds of Trigonella foenum and Coriandrum sativum, which are mixed properly in 1:1 ratio. The antidiabetic activity of the individual plants has been proven. The seed powder of Trigonella foenum showed significant anti hyper glycemic effect against alloxan induced diabetes respectively, in rats at the dose levels of 100 mg/kg [8]. The ethanol extract of the leaves of Coriandrum sativum showed a significant hypo glycemic effect against alloxan induced diabetes in rats at a dose of 200 mg/kg [9].

In the recent era, herbal formulations have gained greater importance than ever before, mainly due to their efficacy and easy availability [10] as well as less side effects as compared to the synthetic drugs [11]. By this advantages have led the people move toward herbal provision, for disease treatment and prevention, and claimed to display synergistic, potential, and agonistic/antagonistic actions and the mixture of species in them shows better therapeutic effect than either species on its own [12]. The theory of poly herbalism has been highlighted in Sharangdhar Samhita, an Ayurvedic literature dating back to 1300 AD [13]. Poly herbal formulations improve the therapeutic action and diminish the concentration of single herbs, in that way reducing the adverse events.

In the diabetic control group, severe body weight loss was observed, which may be due to increased muscle wasting and loss of tissue proteins and insulin deficiency leads to various metabolic alterations in the animals increased blood glucose, increased cholesterol, increased levels of alkaline phosphate and transaminases [14,15]. In the present study, the treatment groups showed significant improvement in body weight, which indicates that the individual extractions, poly herbal formulation and glibenclamide avoid the hyper glycemia induced muscle depletion. The decline in glucose levels may be due to increase in plasma insulin levels or enhanced transport of blood glucose in the peripheral tissue. Our study gives confirmation that the poly herbal formulation enhances the plasma insulin levels and has capable antidiabetic activity [16].

The diabetic hyper glycemia induced by alloxan causes increase of plasma levels of SGPT, SGOT, urea, and creatinine, which are considered as significant markers of liver and renal dysfunction. The poly herbal formulation treated animals reversed the effect of alloxan on the liver and renal markers. This may be due to the hepato protective [17,18] and nephro protective [19,20] mechanism of the individual herbs present in the poly herbal formulation.

Alloxan induced diabetic rats have increased levels of lipid peroxides and reactive oxygen species, which cause hyperglycemia. Incessant generation of free radicals can lead to tissue damage through peroxidation of unsaturated fatty acids [21]. The poly herbal formulation treated animals inhibited the hyperglycemia induced by alloxan, which may be due to the free radical scavenging properties of the individual herbs present in it. Histopathology of the pancreas of alloxan induced diabetic animals showed severely reduce in the number of islets of Langerhans and β cells, with fibrosis and inflammatory cell infiltration into the islets of Langerhans, and these observations are supported by the reports described elsewhere [22]. Poly herbal formulation and glibenclamide treatment to the animals reduced the severity of the histo pathological changes caused by alloxan. 

## Conclusion

Results show the antidiabetic effect of the ethanolic extract of Trigonella foenum seeds, Coriandrum sativum leaves and its poly herbal formulation at the dose of 100 mg/kg. The antidiabetic potential of the polyherbal formulation is comparable with that of glibenclamide, which is shown by decreased levels of blood glucose, total cholesterol, triglyceride, low density lipoprotein (LDL), cholesterol, urea, creatinine, SGOT and SGPT with increase in HDL cholesterol. 

## Figures and Tables

**Table 1:** Effect of EECS and EETF and their poly herbal formulation on bodyweight

			Body weight (gms) (Mean± SEM) on			
S. No	Groups	Treatment	1st day	7th day	14th day	21st day
1	Group-1	2% acacia, 10ml/kg b.w	172.58± 8.47	181.15± 6.71	168.76± 7.11	175.74± 7.06
2	Group-2	Alloxan (150mg/kg, i.p)	154.11± 5.29###	139.87± 11.74###	102.78± 9.87###	79.41±8.28###
3	Group-3	Alloxan (150mg/kg, i.p)and Glibenclamide (0.5 mg/kg, P.O)	164.25± 8.09	158.74± 11.78*	153.78± 12.9***	146.47± 7.09***
4	Group-4	Alloxan (150mg/kg, i.p) ande EETF (100 mg/kg, p.o.),	155.65± 7.36	142.88± 9.48	126.97± 7.22*	84.65± 9.31**
5	Group-5	Alloxan (150mg/kg, i.p) and EECS (100 mg/kg, p.o.)	169.74± 12.74	133.44± 10.42*	124.74± 7.44*	91.78± 9.77**
6	Group-6	Alloxan (150mg/kg, i.p) and EETF + EECS (100 mg/kg, p.o.)	176.84± 7.99	148.47±9.47**	119.47± 10.24***	137.88± 10.24***
All values were expressed as Mean ± S.E.M and n=6; # indicates P<0.05, ## indicates P<0.01, ### indicates P<0.001 when compared to normal group; * indicates P<0.05, ** indicates P<0.01,*** indicates P<0.001 when compared to alloxan induced group (One-way ANOVA followed by Tukey's test)

**Table 2:** Effect of EECS and EETF and their poly herbal formulation on blood glucose levels

			Serum glucose ( mmol/L) (Mean± SEM) on			
S. No	Groups	Treatment	1st day	7th day	14th day	21st day
1	Group-1	2% acacia, 10ml/kg b.w	83.25 ± 3.45	91.25±5.14	86.14± 4.68	89.47± 3.84
2	Group-2	Alloxan (150mg/kg, i.p)	206.25± 9.84###	242.54± 12.57###	294.68± 12.41###	318.66±14.25###
3	Group-3	Alloxan (150mg/kg, i.p) and Glibenclamide (0.5 mg/kg, P.O)	211.74± 11.44	161.74± 9.41*	131.68± 6.74***	97.25± 5.21***
4	Group-4	Alloxan (150mg/kg, i.p) and EETF (100 mg/kg, p.o.),	195.91± 4.58	209.36± 7.25	169.55± 6.35*	134.68± 4.87**
5	Group-5	Alloxan (150mg/kg, i.p) and EECS (100 mg/kg, p.o.)	221.46± 7.09	195.88± 11.25*	157.65± 4.44*	129.84± 6.45**
6	Group-6	Alloxan (150mg/kg, i.p) and EETF + EECS (100 mg/kg, p.o.)	208.67± 7.78	158.74± 9.75**	133.96± 4.82***	99.47± 3.41***
All values were expressed as Mean ± S.E.M and n=6; # indicates P<0.05, ## indicates P<0.01, ### indicates P<0.001 when compared to normal group; * indicates P<0.05, ** indicates P<0.01,*** indicates P<0.001 when compared to alloxan induced group(One-way ANOVA followed by Tukey's test)

**Table 3:** Effect of EECS and EETF and their poly herbal formulation on serum total cholestrol levels

			Serum total cholestrol ( mmol/L) (Mean± SEM) on			
S. No	Groups	Treatment	1st day	7th day	14th day	21st day
1	Group-1	2% acacia, 10ml/kg b.w	69.22± 3.54	68.39± 2.67	71.48± 4.21	70.51± 3.46
2	Group-2	Alloxan (150mg/kg, i.p)	123.35± 4.58##	146.85± 9.47###	169.42± 11.23###	192.37± 10.27###
3	Group-3	Alloxan (150mg/kg, i.p) and Glibenclamide (0.5 mg/kg, P.O)	116.25± 7.71##	123.32± 4.41*	87.36± 5.64***	70.98± 4.21***
4	Group-4	Alloxan (150mg/kg, i.p) and EETF (100 mg/kg, p.o.),	125.36 ± 6.21##	128.22± 6.27	116.44± 6.22*	95.48±9.55**
5	Group-5	Alloxan (150mg/kg, i.p) and EECS (100 mg/kg, p.o.)	113.21± 6.47##	131.24±8.65	111.44±7.21*	89.94±7.36**
6	Group-6	Alloxan (150mg/kg, i.p) and EETF + EECS (100 mg/kg, p.o.)	128.35± 7.32##	113.54±4.57*	105.36± 6.66**	75.98± 7.69***
All values were expressed as Mean ±S.E.M and n=6; # indicates P<0.05, ## indicates P<0.01, ### indicates P<0.001 when compared to normal group; * indicates P<0.05, ** indicates P<0.01,*** indicates P<0.001 when compared to alloxan induced group(One-way ANOVA followed by Tukey's test)

**Table 4:** Effect of EECS and EETF and their poly herbal formulation on serum triglycerides level

			Serum triglycerides ( mmol/L) (Mean± SEM) on			
S. No	Groups	Treatment	1st day	7th day	14th day	21st day
1	Group-1	2% acacia, 10ml/kg b.w	86.37± 2.36	91.24± 4.25	89.35± 4.71	85.94± 4.99
2	Group-2	Alloxan (150mg/kg, i.p)	145.36± 6.47	159.47± 9.44###	167.45± 7.47###	164.21± 11.25###
3	Group-3	Alloxan (150mg/kg, i.p) and Glibenclamide (0.5 mg/kg, P.O)	136.54±6.64	125.35± 6.66*	105.68± 4.25**	91.35± 8.32***
4	Group-4	Alloxan (150mg/kg, i.p) and EETF (100 mg/kg, p.o.),	141.87± 4.25	138.33± 12.22	128.36± 9.97*	118.37± 10.25**
5	Group-5	Alloxan (150mg/kg, i.p) and EECS (100 mg/kg, p.o.)	140.27± 8.06	134.25± 13.36	129.47± 9.64*	125.67± 7.33**
6	Group-6	Alloxan (150mg/kg, i.p) and EETF + EECS (100 mg/kg, p.o.)	132.47± 3.47	121.58± 10.22	115.36± 9.36**	97.25± 6.14***
All values were expressed as Mean ± S.E.M and n=6; # indicates P<0.05, ## indicates P<0.01, ### indicates P<0.001 when compared to normal group; * indicates P<0.05, ** indicates P<0.01,*** indicates P<0.001 when compared to alloxan induced group(One-way ANOVA followed by Tukey's test)

**Table 5:** Effect of EECS and EETF and their poly herbal formulation on serum HDL levels

			Serum HDL ( mmol/L) (Mean± SEM) on			
S. No	Groups	Treatment	1st day	7th day	14th day	21st day
1	Group-1	2% acacia, 10ml/kg b.w	45.36± 2.36	46.35± 3.14	39.47± 4.25	41.25± 2.78
2	Group-2	Alloxan (150mg/kg, i.p)	15.21± 1.24	14.36± 1.03###	15.69± 1.44###	13.36± 0.99###
3	Group-3	Alloxan (150mg/kg, i.p) and Glibenclamide (0.5 mg/kg, P.O)	17.36± 1.14	22.36± 1.66	29.36± 2.19**	37.33± 2.37***
4	Group-4	Alloxan (150mg/kg, i.p) and EETF (100 mg/kg, p.o.)	14.26± 0.99	19.64± 1.11	21.36± 0.97*	26.84± 1.85**
5	Group-5	Alloxan (150mg/kg, i.p) and EECS (100 mg/kg, p.o.)	18.25± 2.21	18.25± 0.74	21.14± 2.07*	29.32± 2.33**
6	Group-6	Alloxan (150mg/kg, i.p) and EETF + EECS (100 mg/kg, p.o.)	20.34± 1.47	25.36± 1.21	28.36± 1.47**	32.7± 2.11***
All values were expressed as Mean ±S.E.M and n=6; # indicates P<0.05, ## indicates P<0.01, ### indicates P<0.001 when compared to normal group; * indicates P<0.05, ** indicates P<0.01,*** indicates P<0.001 when compared to alloxan induced group(One-way ANOVA followed by Tukey's test)

**Table 6:** Effect of EECS and EETF and their poly herbal formulation on serum LDL levels

			Serum LDL ( mmol/L) (Mean± SEM) on			
S. No	Groups	Treatment	1st day	7th day	14th day	21st day
1	Group-1	2% acacia, 10ml/kg b.w	21.36± 0.98	23.14± 1.02	19.35± 1.98	22.74± 0.77
2	Group-2	Alloxan (150mg/kg, i.p)	68.14± 3.58###	74.69± 3.87###	89.47± 5.47###	95.87± 6.64###
3	Group-3	Alloxan (150mg/kg, i.p) and Glibenclamide (0.5 mg/kg, P.O)	68.14± 3.58###	55.68± 3.66*	41.25± 3.47**	27.36± 2.22***
4	Group-4	Alloxan (150mg/kg, i.p) and EETF (100 mg/kg, p.o.),	68.14± 3.58###	65.33± 4.12	55.36± 2.24*	42.36± 2.36**
5	Group-5	Alloxan (150mg/kg, i.p) and EECS (100 mg/kg, p.o.)	68.14± 3.58###	61.25± 3.55	51.28± 3.21*	39.11± 4.22**
6	Group-6	Alloxan (150mg/kg, i.p) and EETF + EECS (100 mg/kg, p.o.)	68.14± 3.58###	55.47± 3.47	45.17± 4.11**	32.77± 3.64***
All values were expressed as Mean ±S.E.M and n=6; # indicates P<0.05, ## indicates P<0.01, ### indicates P<0.001 when compared to normal group; * indicates P<0.05, ** indicates P<0.01,*** indicates P<0.001 when compared to alloxan induced group(One-way ANOVA followed by Tukey's test)

**Table 7:** Effect of EECS and EETF and their poly herbal formulation on serum VLDL levels

			Serum VLDL ( mmol/L) (Mean± SEM) on			
S. No	Groups	Treatment	1st day	7th day	14th day	21st day
1	Group-1	2% acacia, 10ml/kg b.w	16.35± 0.78	18.24± 1.21	21.21± 0.75	18.98± 0.89
2	Group-2	Alloxan (150mg/kg, i.p)	42.25± 2.36##	49.58± 5.47###	54.69± 4.98###	65.47± 6.43###
3	Group-3	Alloxan (150mg/kg, i.p) and Glibenclamide (0.5 mg/kg, P.O)	41.36± 4.32##	36.14± 3.14*	29.47± 2.21**	24.25± 1.79***
4	Group-4	Alloxan (150mg/kg, i.p) and EETF (100 mg/kg, p.o.),	38.32± 3.47##	35.65± 3.21	33.25± 1.25**	30.21± 2.57**
5	Group-5	Alloxan (150mg/kg, i.p) and EECS (100 mg/kg, p.o.)	51.25± 4.25##	45.26± 5.34	41.25± 3.84**	33.48± 3.47**
6	Group-6	Alloxan (150mg/kg, i.p) and EETF + EECS (100 mg/kg, p.o.)	45.58± 3.36##	41.87± 5.47*	37.48± 4.44**	28.67± 2.35***
All values were expressed as Mean± S.E.M and n=6; # indicates P<0.05, ## indicates P<0.01, ### indicates P<0.001 when compared to normal group;
* indicates P<0.05, ** indicates P<0.01,*** indicates P<0.001 when compared to alloxan induced group(One-way ANOVA followed by Tukey's test)

**Table 8:** Effect of EECS and EETF and their poly herbal formulation on hepatic and renal biomarkers

S. No	Groups	Treatment	SGOT	SGPT	Urea	Creatinine
1	Group-1	2% acacia, 10ml/kg b.w	89.47± 3.41	79.55± 4.08	21.21± 1.75	0.55± 0.07
2	Group-2	Alloxan (150mg/kg, i.p)	167.84± 11.33###	174.39± 15.31###	61.42± 5.48###	1.98± 0.15###
3	Group-3	Alloxan (150mg/kg, i.p) and Glibenclamide (0.5 mg/kg, P.O)	96.35± 7.08***	93.74± 7.09***	28.94± 3.41**	0.72± 0.04***
4	Group-4	Alloxan (150mg/kg, i.p) and EETF (100 mg/kg, p.o.),	127.47± 7.36***	147.36± 4.69*	42.98± 2.81**	1.12± 0.06**
5	Group-5	Alloxan (150mg/kg, i.p) and EECS (100 mg/kg, p.o.)	132.78±6.75*	132.99± 7.09**	46.87±5.88**	1.09± 0.12**
6	Group-6	Alloxan (150mg/kg, i.p) and EETF + EECS (100 mg/kg, p.o.)	112.47± 7.94***	102.47± 6.44***	35.62± 2.47***	0.79± 0.05***
All values were expressed as Mean ± S.E.M and n=6; # indicates P<0.05, ## indicates P<0.01, ### indicates P<0.001 when compared to normal group; * indicates P<0.05, ** indicates P<0.01,*** indicates P<0.001 when compared to alloxan induced group(One-way ANOVA followed by Tukey's test)

## References

[1] Huai H (2010). Ethnobot Res Appl.

[2] Husain SZ (2008). Pak J Bot.

[3] Adeghate E (2006). Ann N Y Acad Sci.

[4] Parasuraman S (2010). Journal of pharmacology and Pharmacotherapeutics..

[5] Mandavi (2012). Indian J Med Res.

[6] https://www.who.int/.

[7] Katsumata K (1992). Horm Metab Res.

[8] Mowl A (2009). African Journal of Traditional, Complementary and Alternative Medicines.

[9] Sreelatha S, Inbavalli R (2012). Journal of Food Science..

[10] Petchi RR (2014). Journal of Traditional and Complementary Medicine.

[11] Sen A (2011). J Cell Biol.

[12] Sujatha S, Shalin JJ (2012). Asian J Sci Res.

[13] Srivastava S (2012). Phytopharmacology.

[14] Szkudelski T (2001). Physiological Research..

[15] Yadav S (2002). Journal of Ethnopharmacology..

[16] Wilcox G (2005). Clin Biochem Rev.

[17] Kaviarasan S, Anuradha CV (2007). Die Pharmazie - An International Journal of Pharmaceutical Sciences.

[18] Pandey A (2011). Journal of Pharmacy and Bioallied Sciences..

[19] USLU GA (2019). Biomedical Research and Therapy.

[20] Lakhera A (2015). Interdisciplinary Toxicology.

[21] Kumar V (2013). BMC complementary and Alternative Medicine.

[22] Abdollahi M (2011). Histology and Histopathology.

